# Quality of life and symptoms before and after nasal septoplasty compared with healthy individuals

**DOI:** 10.1186/s12901-016-0031-7

**Published:** 2016-10-28

**Authors:** Vegard Bugten, Ann Helen Nilsen, Wenche Moe Thorstensen, Mads Henrik Strand Moxness, Marit Furre Amundsen, Ståle Nordgård

**Affiliations:** 1 Department of Neuroscience, Faculty of Medicine, NTNU, Norwegian University of Science and Technology, 7006 Trondheim, Norway; 2 Department of Ear, Nose and Throat, Head and Neck Surgery, St. Olavs Hospital, Trondheim, Norway

**Keywords:** Nasal septoplasty, SNOT, Allergic rhinitis, Allergy, Snoring, Obstructive sleep apnea, Chronic rhinosinusitis

## Abstract

**Background:**

The goal of this study is to compare quality of life (Qol) and symptoms in 91 patients with a deviated nasal septum preoperatively and postoperatively with a control group of 93 healthy individuals.

**Methods:**

All patients reported Qol on Sino-Nasal-Outcome-Test-20 (SNOT-20) and symptoms on visual analogue scale (VAS) preoperatively and 6 months after surgery and the results were compared with the controls.

**Results:**

Mean SNOT-20 score improved from 1.8(SD0.9) preoperatively to 0.9(SD0.8) postoperatively (*p* < 0.000) but did not reach the same level as the controls 0.4(SD0.5). Septum surgery leads to a significant symptom improvement for all symptoms investigated (*p* < 0.000) on VAS. The patients reached the same level as the healthy controls in 6 of 11 symptoms (headache, facial pain, sneezing, trouble with rhinosinusitis, cough and snoring) but the patients group had significantly more trouble with nasal blockage (VAS 29 vs 9), change in sense of smell (VAS 12 vs5), nasal discharge (VAS 22 vs 11), oral breathing (VAS 23 vs 13) and reduced general health (VAS 12 vs 5) also postoperatively (*p* < 0.01). Sub analyses showed that allergic patients reported a VAS score of 36 (SD30) for nasal blockage and 17 (SD22) for facial pressure postoperatively versus 23(SD22) and 6(SD13) in non-allergic patients (*p* < 0.03 and *p* < 0.01). Patients with obstructive sleep apnea syndrome (OSAS) reported more trouble with snoring on VAS postoperatively than other patients, 42(SD28) versus 20(SD23) (*p* < 0.002).

**Conclusion:**

Septoplasty leads to a highly significant improvement in Qol and symptoms. The patients do not reach the same level of Qol as healthy controls. All symptoms are reported as mild on VAS postoperatively.

Allergic patients tend to report more nasal blockage and facial pressure postoperatively than other patients and a focus on medical treatment should be kept also postoperatively. Patients with obstructive sleep apnea report more trouble with snoring postoperatively and alterative treatment options for snoring may be considered in these patients.

**Electronic supplementary material:**

The online version of this article (doi:10.1186/s12901-016-0031-7) contains supplementary material, which is available to authorized users.

## Background

Nasal blockage is a common complaint. In the adult population chronic diseases such as chronic rhinosinusitis and allergic rhinitis are two of the main causes for nasal congestion. In Europe, chronic rhinosinusitis has a prevalence of 10,9 % [1] and allergic rhinitis a prevalence of 17–29 % [2]. These diseases cause mucosal congestion and lead to nasal obstruction with reduced nasal airflow. Other causes for nasal blockage may be structural, where variations of the cartilaginous and bony structures of the nose can lead to problems with reduced nasal airflow. Nasal septal deviations have a prevalence ranging from 19 to 65 % due to different criteria for defining a deviated septum [3, 4].

Some patients with a deviated septum need surgery to relieve symptoms. Nasal septoplasty is the third most common surgery [5] performed in the ear nose and throat (ENT) specialty and can be done under local or general anesthesia. Nevertheless, the gain of this surgery has been questioned. Some studies have showed moderate to excellent results [6, 7] but a new study from Sweden suggests that the results are unsatisfactory [8]. Additionally, radiofrequency therapy of the inferior turbinate is done frequently as an attempt to reduce nasal congestion or hypertrophy of the inferior turbinate [9]. Other procedures to reduce the size of the inferior turbinate turbinate can be complete or partly resection of the turbinate or use of instruments as the microdebriders, laser or bipolar diathermy.

In 2012 our department started a quality register to evaluate the results of nasal surgery. The scope of the register is to evaluate our results after existing or new surgical procedures or measures and hence improve daily praxis and treatment plans to the best for our patients.

The primary goal of this study is to compare the Qol and symptoms in patients undergoing septoplasty with or without surgery of the inferior turbinate with Qol and symptoms in healthy individuals. Secondary we evaluate the effect of surgery on QoL and symptoms in the patient group.

## Materials and methods

### Ethics, consent and permissions

This prospective study has been conducted during the period from January 2012 to April 2015 and is approved by the Committee for Medical Research Ethics in Norway, 2015/367/REK NORD. All patients and controls signed a written consent prior to inclusion in the trial.

## Materials

All patients were referred to the ENT department at St Olav’s Hospital/Trondheim University hospital from general practitioners, ENT specialists or other hospitals in the region. All patients were examined at the outpatient clinic by a variety of surgeons. The diagnosis was based on endoscopic examination of the nose combined with patients’ symptoms. When there was indication for septoplasty alone or septoplasty combined with radiofrequenzy therapy of the inferior turbinate, the patients were asked to participate in the study. Inclusion criteria were a deviated nasal septum with nasal breathing problems. Exclusion criteria were age less than 18 years and language problems with difficulty to interpret the questionnaires.

We included 103 patients with septal deviation. Twelve patients did not attend for surgery. Thus 91 patients underwent surgery and 86 came for the postoperative control 6 months after surgery. Ninety three healthy individuals without nasal complaints were used as controls.

## Methods

### Quality of life and symptoms preoperatively and 6 months postoperatively

Quality of life was assessed with the Sino-Nasal-Outcome-Test-20 (SNOT 20) questionnaire which is a validated disease specific Qol questionnaire for rhino-sinusitis [10, 11]. The patients graded 20 symptoms on a scale from 0 (no symptoms) to 5 (problem as severe as can be). The SNOT score for each patient was defined as the mean value of the response to the 20 items. The questionnaire is divided into 4 subsets [12]. The first subset includes symptoms related to nose, the second subset focuses on ear and face, the third subset considers sleep quality and the fourth subset has questions about psychological issues.

Symptoms were reported on 100 mm visual analog scales (VASs) where 0 mm represents no symptoms and 100 mm represent “as troublesome as possible”. Symptoms reported are nasal obstruction, headache, facial pain, facial pressure, reduced sense of smell, nasal discharge, sneezing, cough, snoring, and oral breathing and reduced general health. The symptom severity is considered mild between 0 and 30, moderate from 30 to 70 and severe from 70- 100 [13].

### Patient satisfaction

The patients were asked if they were satisfied with the results of surgery 6 months after surgery. They answered the question with yes or no.

### Statistics

Descriptive results are presented as mean with standard deviation (SD) or range. We used exact test to compare ordinal and categorical variables. In the statistical analyses we used paired samples T test to compare mean values preoperatively and postoperatively in the patient group. To compare mean values in the control group with the mean postoperative values in the patient group, we used independent samples T test. In additional analyses, we also used a general linear model to adjust for potential differences in the distribution of age and sex in the groups.

We have done power calculations. It showed that with 40 patients in each group and a significance level of 0.05(alpha), we were able to detect a difference in SNOT-20 of 0.6 (SD 1.2) between the groups with 80 % power. With 100 participants in each group and the same assumptions as above, we were able to detect a difference in SNOT-20 of 0.4.

All statistical analysis was performed using PASW Statistics, version 22 for Windows (SPSS Inc., Chicago, Illinois) and a two—sided *p*-value less than 0.05 was considered significant.

## Results

Baseline information of the patients and control group are given in Table 1.

**Table 1:** Demographic data on the patient and control groups

	Septoplasty (*n* = 91)	Control group (*n* = 93)
Mean age (range)	39 (18–68)	43.8 (20–65)
Mean BMI, kg/m^2^	27.3 (5)	25.3 (3.4)
Male/female	75/16	41/52
Airborne allergy Pollen Dust mite Animal hair	36/91 30/36 14/36 20/36	20/93
Asthma	14/91	0
Smoking	18/91	12
Previous surgery in the nose Septoplasty Sinus surgery because of CRS Sinus surgery because of NP Coblation of inferior turbinate	24/91 13/24 3/24 5/24 6/24	0
Obstructive sleep apnea Mean AHI (SD)	15/91 22.9 (19)	

### Surgical procedure and postoperative care

Twenty one patients underwent surgery under local anesthesia and 70 had general anesthesia. Average time for the surgery was 76 min (range 10–153). Fifty-one patients underwent traditional cartilage preserving septoplasty alone and 40 patients had a combination of septoplasty and radiofrequency therapy of the inferior tubinates. The procedures were done by 13 different surgeons, 6 consultants and 7 senior registrars. Seventy-seven (77/91) had a silastic plate bilaterally for support and to prevent adhesions postoperatively. Forty-three patients had a nasal packing for approximately 2 days postoperatively to prevent bleeding and septum hematoma. The nasal packing was removed by a nurse in the outpatient clinic or by the patients themselves. The plates were taken out approximately 1 week after surgery by the surgeon.

### Sino Nasal Outcome Test (SNOT-20) before- and 6 months after surgery

SNOT 20 showed a significant reduction for all symptoms included in the test (Table 2). Average SNOT-20 score was 1.8 (SD 0.9) preoperatively and 0.9 (SD 0.80) postoperatively (*p* < 0.000). We found highly significant improvements for all four subsets. Sleep function seemed to improve better than the items from the nose and psychological subsets. The items from the subset of ears and face had a smaller absolute improvement than the other subsets but also this subset improved significantly after surgery. When we compared the postoperative SNOT score from the patients with the SNOT score in the healthy control group, we found that the control group had less trouble with most of the items in the questionnaire. Nevertheless, it was interesting to find no significant differences between the patients and healthy controls in the Ear and face subset of the SNOT-20 questionnaire postoperatively (Table 2). In additional multivariable analyses, we also adjusted for age and sex, but this did not change the results.

**Table 2:** presents mean values on SNOT 20 in the patient group and in the control group

Question	Mean pre-operative score	Mean post-operative score	*P*-value^A^	Mean score Control group	*P* Value^B^
Need to blow nose^a^	2.8(1.3)	1.7(1.3)	0.000	0.6(0.8)	0.000
Sneezing^a^	1.77(1.3)	1.0(1.0)	0.000	0.5(0.7)	0.000
Runny nose^a^	2.0(1.5)	1.0(1.1)	0.000	0.4(0.7)	0.000
Cough	1.2(1.2)	0.7(0.9)	0.000	0.3(0.7)	0.005
Postnasal discharge^a^	1.6(1.4)	0.9(1.3)	0.000	0.2(0.6)	0.000
Thick nasal discharge^a^	1.9(1.5)	1.0(1.3)	0.000	0.2(0.5)	0.000
Ear fullness^b^	1.2(1.3)	0.7(1.1)	0.000	0.6(1.1)	0.4
Dizziness^b^	1.0(1.2)	0.4(0.7)	0.000	0.4(0.8)	0.4
Ear pain^b^	0.7(1.1)	0.4(0.9)	0.01	0.4(0.9)	0.9
Facial pain/pressure^b^	1.2(1.4)	0.4(0.9)	0.000	0.2(0.5)	0.05
Difficulty falling to sleep^c^	1.7(1.5)	1.0(1.3)	0.000	0.3(0.9)	0.000
Wake up at night^c^	2.5(1.5)	1.3(1.3)	0.000	0.7(1.2)	0.008
Lack of good night sleep^c^	2.8(1.6)	1.5(1.5)	0.000	0.6(1.2)	0.000
Wake up tired	3.0(1.3)	1.7(1.5)	0.000	1.0(1.8)	0.01
Fatigue^d^	1.8(1.5)	0.9(1.3)	0.000	0.3(0.7)	0.000
Reduced productivity^d^	1.9(1.1)	0.9(1.1)	0.000	0.2(0.6)	0.000
Reduced consentration^d^	2.1(1.4)	1,0(1.2)	0.000	0.4(0.7)	0.000
Frustrated/restless/ irritable^d^	2.0(1.6)	0.9(1.3)	0.000	0.3(0.7)	0.003
Sad^d^	1.0(1.3)	0.5(1.0)	0.000	0.2(0.5)	0.003
Embarrassed^d^	0.8(1.0)	0.3(0.8)	0.001	0.1(0.3)	0.007
Subset					
Rhinologic^a^	1.9(1.0)	1.0(0.8)	0.001	0.4(0.4)	0.000
Ear/face^b^	1.0(1.0)	0.5(0.7)	0.000	0.4(0.7)	0.2
Sleep function^c^	2,3(1.3)	1.3(1.3)	0.000	0.5(0.9)	0.000
Psycological function^d^	1.6(1.1)	0.7(1.0)	0.000	0.3(0.5)	0.000
Mean SNOT-20 score	1.8(0.8)	0.9(0.8)	0.000	0.4(0.5)	0.000

*P*- value^A^ is the p value for improvement in the patient group from preoperatively to postoperatively. *P*-value^B^ is p-value for a difference between postoperative SNOT-20 in the patients compared to the control group

^a^ Questions belonging to rhinologic subset

^b^ Questions belonging to ear/facial subset

^c^ Questions belonging to sleep function subset

^d^ Questions belonging to psychological function subset

### Symptoms on visual analog scales (VASs) before and 6 months after surgery

Regarding symptoms given on VAS we found that septum surgery leads to a significant symptom improvement for all symptoms investigated (Table 3). We compared postoperative symptoms in the patient group with symptoms in the control group and found that the patients group had significantly more trouble with nasal blockage, change in sense of smell, nasal discharge, oral breathing and reduced general health also postoperatively (Table 3). Additional multivariable analyses with adjustments for age and sex were done without change in the results. We also did sub analyses, where patients who have had septoplasty alone where compared with those who had septoplasty combined with radiofrequency therapy of the inferior turbinate. No significant differences in SNOT scores and postoperative symptoms between the groups were shown.

**Table 3:** Symptoms on VAS in the patient group and the control group

Symptoms	Mean Preoperatively VAS (SD )	Mean Postoperatively VAS (SD )	*P*-value_1_	Control group	*P*-value_2_
Nasal blockage	71(SD20)	29(SD26)	0.000	9 (11)	0.000
Headache	33(SD30)	18(SD25)	0.000	16 (20)	0.5
Facial pain	16(SD24)	7(SD14)	0.000	9 (17)	0.4
Altered sense of smell	31(SD32)	12(SD24)	0.000	5 (9)	0.009
Nasal discharge	42(SD32)	22(SD29)	0.000	11 (19)	0.003
Sneezing	32(SD28)	17(SD23)	0.000	14 (15)	0.3
Trouble with rhinosinusitis	16(SD28)	4(SD12)	0.000	3 ( 7)	0.9
Cough	22(SD27)	9(SD17)	0.000	7 ( 9)	0.3
Snoring	54(SD33)	26(SD26)	0.000	26 (29)	0.8
Oral breathing	63(SD30)	23(SD27)	0.000	13 (22)	0.01
Reduced general health	47(SD31)	12(SD20)	0.000	5 ( 6)	0.007

*P*-value_1_ is the p-value for improvement from preoperatively to postoperatively. *P*-value _2_ is the p-value for a difference in postoperative symptoms in the patients compared to the controls

### Allergy

We compared mean postoperative total SNOT-20 values in the allergic patients (1.0 (SD 0.9)) with non-allergic patients (0.9 (SD 0.7)) and found no significant differences (*p* = 0.47). We also compared preoperative and postoperative symptoms in allergic and non-allergic patients. Preoperatively there were no differences between the groups but postoperatively the allergic patients were significantly more bothered with nasal blockage (*p* = 0.03) and facial pressure (*p* = 0.01) than the non-allergic patients. The allergic patients reported a mean value of 36 (SD30) on VAS for nasal blockage postoperatively and the non-allergic patients reported 23 (SD22) on VAS. For facial pressure the values on VAS were 17 (SD26) in allergic versus 6 (SD13) in the non-allergic patients.

### Asthma

Mean postoperative total SNOT-20 value in the fourteen asthma patients was 1.0 (SD 0.8) versus 0.9 (SD 0.8) in the non- asthmatic patients (*p* < 0.55). We also compared postoperative symptoms in asthmatic and non-asthmatic patients and found no significant differences between the groups postoperatively.

### Obstructive sleep apnea syndrome (OSAS)

Fifteen patients reported they had obstructive sleep apnea syndrome (OSAS) preoperatively. We compared mean postoperative total SNOT-20 values in OSAS patients and non-OSAS patients and found no significant differences (*p* = 0.18). Mean values in the OSAS patients were 1.0 (SD 0.8) and 0.9 (SD 0.8) in patients without OSAS. We also compared postoperative symptoms in patients with OSAS with patients without OSAS. We found that OSAS patients report significantly more trouble with snoring postoperatively than other patients (*p* < 0.002). The OSAS patients reported a mean VAS score for snoring of 40 (SD 29) postoperatively compared to a mean VAS score of 20 (SD 24) in the other patients after surgery.

### Chronic rhinosinusitis (CRS)

Eight patients had earlier undergone sinus surgery because of CRS. We compared the SNOT score and symptoms in these patients with the other patients and found that the CRS patients reported significantly more trouble in the ear and face segment of SNOT-20. Preoperatively they reported 1.9(SD1.0) versus 0.9(SD0.9) (*p* < 0.01) in the other patients and postoperatively 1.2(SD1.1) versus 0.4(SD0.8) (*p* < 0.05). Regarding the symptoms on VAS, patients with previous surgery for CRS had significantly more trouble with rhinosinusitis than the other patients had preoperatively and postoperatively. Preoperatively VAS score for rhinosinusitis was 41(SD35) versus13 (SD25) in the other patients, postoperatively the scores changed to 33(SD34) versus 10(SD24). For all the other symptoms we found no differences.

### Gender

We found no significant difference between men and women regarding mean pre- and postoperative SNOT-20 (*p* = 0.1 and *p* = 0.78). Evaluating the VAS scores pre and postoperatively we found that the women in this study report more symptoms preoperatively than the men but postoperatively there were no significant differences between men and women. The women reported significantly (*p* < 0.05) more trouble with nasal blockage, facial pressure, sneezing, and reduced general condition than the men. Average VAS score for all symptoms in women was 47 (SD15) preoperatively and average VAS score in men was 35(SD18). The difference was significant preoperatively (*p* < 0.02). Postoperatively average VAS score was 14 (SD15) in both groups.

### Patient satisfaction 6 months after surgery

Six months after surgery we asked the patients if they were satisfied with the result. Sixty-two patients (62/86) answered the question. Forty-seven (47/62) were satisfied (76 %) and fifteen (15/62) were not (24 %). We found that previous septoplasty patients had significantly greater risk of being unsatisfied with the result after surgery (*p* < 0.028). Six patients (6/13) with previous septoplasty were not satisfied with the result 6 months after surgery (46 %). Eight of the patients (8/51) without previous surgery were not satisfied with the result 6 months after surgery (16 %). We compared symptoms preoperatively and postoperatively on VAS and found that patients who been operated previously reported significantly (*p* < 0.05) more trouble with headache (28 vs 15), facial pain (13 vs 4), nasal secretions (35 vs 17), mouth breathing (36 vs 23) and reduced general condition (20 vs 9) postoperatively than the other patients.

## Discussion

This study shows that nasal septoplasty leads to a better sino-nasal Qol and a significant improvement for all symptoms given on VAS. The patients do not reach the same Qol postoperatively as healthy controls. The patient group has a significantly lower sino-nasal Qol in three of four subsets in SNOT-20 postoperatively. Regarding symptoms on VAS, the patients reach the same level as the healthy controls in 6 of 11 symptoms. For symptoms as nasal blockage, change in sense of smell, nasal secretions, oral breathing and reduced general condition we find a greater symptom burden in the patient group postoperatively.

The patients report a mean preoperative SNOT-20 score of 1.8. Comparing the preoperative results from the SNOT-20 questionnaire with results from other studies we see that the patients with septal deviations report Qol at approximately the same level as patients with CRS [11] and a worse SNOT-20 score than asthmatic patients who report a mean SNOT-20 score of 1.3 [14].

Nasal septal surgery leads to a highly significant improvement in mean SNOT-20 score. For all subsets in the questionnaire we find a highly significant improvement. When comparing the postoperative results with healthy controls we find that the patients reach the same level as the controls in the ear and face subset of the questionnaire where the symptom burden is comparable in the groups. For the rhinologic -, sleep function-, and the psychological subset there is a difference between the groups postoperatively. The average improvement on SNOT-20 was 0.9. Piccirillo et al report that a reduction on SNOT-20 equal 0.8 or better is regarded as clinical significant [11]. Anyway, the patient group reaches a mean SNOT-20 score of 0.9 postoperatively which represent a place between no symptoms and very mild symptoms on the scale from zero to five.

Looking closer at the symptom scores given on VAS we find that surgery has a highly significant effect on all symptoms and it is confirmed that the main symptom in patients with nasal septal deviations is nasal blockage. The nasal blockage in the patient group may enhance symptoms such as snoring, oral breathing and nasal discharge, which consequently may reduce the general health of the patients. Surgery leads to a highly significant symptom improvement. Postoperatively, the patients reach the same symptom level as the healthy controls in 6 of 11 symptoms, and all symptoms were reported as mild on VAS postoperatively [13].

Baseline data show that some of the patients who undergo nasal septoplasty have coexisting diseases such as allergy, asthma, obstructive sleep apnea and CRS. These are all conditions which may be influenced by nasal blockage. It is easy to accept that allergic rhinitis together with septal deviations can lead to more symptoms from the nose than these two conditions separately. The fact that 40 % of the patients report that they have allergic rhinitis support this assumption; allergy and septal deviations together render the patients motivated for surgery. When we did sub analyses where we compared Qol and postoperative symptoms in allergic and non-allergic patients we found no differences between the groups on SNOT-20, but regarding nasal blockage and facial pressure we found that the allergic patients reported more symptoms on VAS than non-allergic patients postoperatively. This difference is probably caused by nasal congestion due to allergy and is important to acknowledge by the surgeon and the patients. Allergic patients might not respond as well as non-allergic patients to septoplasty and they might profit from additional preoperative investigations, for instance CT of the nose and sinus, to improve the planning of the surgery. Nevertheless, it is of great importance that the allergic patients are treated medically also postoperatively to optimize the outcome after surgery.

It is shown in an earlier study that asthmatic patients have more symptoms of nasal blockage than non-asthmatic patients [14] and that they need an open nose to optimize the air for the lower airways. A blocked nose with the consequent lack of cleaning, humidification and warming of the inspired air [15] may harm the lungs and lead to more troublesome asthma. Therefore, treatment of nasal blockage in these patients is even more important. We did sub analyses on the fifteen asthmatic patients (15/95) in this study where we compared Qol and postoperative symptoms on VAS in asthmatic patients with patients without asthma. We found no significant differences postoperatively indicating that asthma patients reach the same QOL and symptom level as the other patients.

Snoring is considered problematic among our patients. The sleep subset in SNOT-20 shows that patients with indication for a septoplasty have problems with sleep quality. Fifteen patients (15/91) reported they have obstructive sleep apnea. Twelve of these patients had trouble using or trouble accepting the continuous positive airway pressure (CPAP) due to nasal blockage. After surgery the patients with OSAS report improvement for all symptoms on VAS but when we look separately at snoring on VAS we find that the OSAS patients have significantly more trouble with snoring postoperatively than the other patients. This may be explained by a different anatomic configuration of the soft palate or oral cavity that may lead to breathing problems during sleep. Unfortunately, we have no documentation to prove this.

Many patients with CRS have a deviated nasal septum. In this study, eight patients had previously undergone sinus surgery with unsatisfactory results. Sub analyses of the symptoms on VAS and SNOT-20showed that the CRS patients responded similar to nasal septum surgery as the other patients but report more trouble with rhinosinusitis preoperatively and postoperatively and more trouble in the ear and face segment of SNOT 20. This is comprehensible because symptoms of rhinosinusitis is more expected in CRS patients than patients with a deviated nasal septum.

We see that more men than women undergo septum surgery. The reason for this is not clear, but we think that men tend to be involved in activities that are associated with a higher risk for nasal traumas. Nevertheless, both men and women respond to nasal septum surgery and reach the same Qol and symptom level postoperatively.

The patients were asked if they were satisfied with the result after surgery. 76 % answered yes and 24 % answered no. This corresponds with other studies [6]. We did sub analyses and found that 86 % of the patients who underwent surgery for the first time were satisfied but only 54 % of the patients who had undergone previous nasal septoplasty were satisfied. Patients who had undergone nasal surgery previously also reported more symptoms on VAS postoperatively. This indicates that revision surgery is more difficult and extra caution and exact planning is paramount when doing revision nasal septoplasty.

The major strengths of this study are the pragmatic study design based on prospective registry data in an everyday clinical setting. All patients selected for surgery were asked to participate. Eighty-seven of 91 patients who underwent surgery came for the postoperative control and all patients were at the out patient’s clinic when they reported their symptoms which should minimize errors in their reporting.

This study has some limitations. We have not randomized the patients to other treatment options for comparison. The use of SNOT-20 as a tool to assess sino-nasal Qol has some limitations due to lack of questions about nasal obstruction and loss of smell and taste. However, we compensate for this by evaluating nasal obstruction and change of smell on VAS.

## Conclusion

In this study we have shown that nasal septoplasty leads to a better sino-nasal Qol and a highly significant symptom improvement. The patients do not reach the same Qol as healthy controls but they reach the same symptom level in six of eleven symptoms. In average all symptoms are reported as mild on VAS. Allergic patients tend to have more trouble with nasal blockage and facial pressure postoperatively than other patients and should therefore have focus on medical treatment also postoperatively. Patients with obstructive sleep apnea syndrome report more trouble with snoring postoperatively than the other patients and alternative treatment options for snoring may be considered in these patients.

## Additional files

Additional file 1: Spreadsheet that include information from the patients about Qol and symptoms given in the SNOT-20 questionnaire and on VASs. (XLSX 51 kb)
Additional file 2: Spreadsheet that include information from the control group of healthy persons about Qol and symptoms given in the SNOT-20 questionnaire and on VASs. (XLS 58 kb)

## Abbreviations

AHI: Apnea hypopnea index
CPAP: Continuous positive airway pressure
CRS: Chronic rhinosinusitis
NP: Nasal polyps
OSAS: Obstructive sleep apnea syndrome
Qol: Quality of life
SD: Standard deviation
SNOT-20: Sino-Nasal-Outcome-Test-20
VAS: Visual analogue scale
vs: Versus
